# Climate Extremes and the Length of Gestation

**DOI:** 10.1289/ehp.1003241

**Published:** 2011-06-09

**Authors:** Payam Dadvand, Xavier Basagaña, Claudio Sartini, Francesc Figueras, Martine Vrijheid, Audrey de Nazelle, Jordi Sunyer, Mark J. Nieuwenhuijsen

**Affiliations:** 1 Centre for Research in Environmental Epidemiology (CREAL), Barcelona, Spain; 2 Municipal Institute of Medical Research (IMIM-Hospital del Mar), Barcelona, Spain; 3 CIBER Epidemiología y Salud Pública (CIBERESP), Spain; 4 Department of Maternal-Fetal Medicine, ICGON (Institut Clínic d´Obstetrícia, Ginecologia i Neonatologia), Hospital Clinic-IDIBAPS (Institut d´Investigacions Biomèdiques Agustí Pi i Sunyer), University of Barcelona, Barcelona, Spain; 5 Pompeu Fabra University, Barcelona, Spain

**Keywords:** climate, climate change, gestational age, global warming, hot temperature, perinatal mortality, pregnancy outcome, preterm birth

## Abstract

Background: Although future climate is predicted to have more extreme heat conditions, the available evidence on the impact of these conditions on pregnancy length is very scarce and inconclusive.

Objectives: We investigated the impact of maternal short-term exposure to extreme ambient heat on the length of pregnancy.

Methods: This study was based on a cohort of births that occurred in a major university hospital in Barcelona during 2001–2005. Three indicators of extreme heat conditions based on 1-day exposure to an unusually high heat–humidity index were applied. Each mother was assigned the measures made by the meteorological station closest to maternal residential postcodes. A two-stage analysis was developed to quantify the change in pregnancy length after maternal exposure to extreme heat conditions adjusted for a range of covariates. The second step was repeated for lags 0 (delivery date) to 6 days.

Results: We included data from 7,585 pregnant women in our analysis. We estimated a 5-day reduction in average gestational age at delivery after an unusually high heat–humidity index on the day before delivery.

Conclusion: Extreme heat was associated with a reduction in the average gestational age of children delivered the next day, suggesting an immediate effect of this exposure on pregnant women. Further studies are required to confirm our findings in different settings.

Gestational age at delivery is one of the key determinants of fetal maturity at birth. Reduction in gestational age at delivery is reported to be the leading cause of perinatal mortality in the United States and Europe, and the risk increases progressively as gestational age at birth declines (Berkowitz and Papiernik 1993; Goldenberg et al. 2008; Morrison et al. 1995; Zanardo et al. 2004). Even a 1-week reduction from term in gestational age at delivery has been shown to adversely affect perinatal outcomes (Morrison et al. 1995; Zanardo et al. 2004). Shortened pregnancy has also been increasingly related to adverse health outcomes in later life, including neurodevelopmental disorders, bronchopulmonary dysplasia, and growth impairment (Berkowitz and Papiernik 1993; Gibson 2007). The reduction in gestational age at delivery has been suggested to have a multifactorial origin, including fetomaternal and environmental factors (Moutquin 2003). A growing body of evidence has linked environmental insults such as maternal stress, infection, smoking, socioeconomic factors, nutrition, psychosocial factors, and pollution to the shortening of gestation (Goldenberg et al. 2008; Murphy 2007; Steer 2005).

Heat stress is a function of the interaction of internal heat production, capacity for heat loss to the environment, and environmental heat load (Wells and Cole 2002). During pregnancy, increases in fat deposition and decreases in the ratio of body surface area to body mass due to weight gain result in less capacity for heat loss to the environment (Falk 1998; Wells 2002; Wells and Cole 2002). There is also an increase in internal heat production due to fetal growth and metabolism (Wells 2002; Wells and Cole 2002). These all limit the ability of pregnant women to mitigate heat stress and make them more prone to heat stress due to environmental heat load (Wells and Cole 2002).

The pattern of climate extremes has been considerably changed during the second half of the 20th century (Frich et al. 2002). The future climate is predicted to have “more intense, longer lasting and/or more frequent” extreme heat episodes (Meehl and Tebaldi 2004). These extreme heat conditions have been shown to affect pregnancy outcomes in animals, including shortening of pregnancy (Dreiling et al. 1991; Rensis and Scaramuzzi 2003). However, the available evidence on the impact of this exposure on human pregnancy is very scarce. An earlier American study by Lajinian et al. (1997) investigated the association between an increased heat–humidity index and rate of preterm labor among patients of a municipal hospital in Brooklyn during 2 summer and 2 winter weeks (Lajinian et al. 1997). Although they reported an association, their study was based on a small number of preterm births (75 cases) and the analysis was not adjusted for possible confounders (Lajinian et al. 1997). That study was followed by another American study (Porter et al. 1999) based on Illinois vital record files that detected no association between maternal exposure to short-term heat stress and pregnancy length after adjusting for maternal ethnicity, academic level, and socioeconomic status. That study classified gestational age based on the date of the last menstrual period reported by the mothers and used meteorological data from one monitoring station to classify exposures for the entire city of Chicago. Both studies called for further research. Our study aimed to investigate the impact of maternal short-term exposure to extreme ambient heat on the length of pregnancy.

## Materials and Methods

*Study setting and data sources.* This study was based on a cohort of births that occurred between January 2001 and June 2005 in the obstetrics department of the Hospital Clinic of Barcelona, a major university hospital covering the city of Barcelona (Spain), with a catchment area of about one million inhabitants (Figueras et al. 2008). The database included a wide range of prospectively collected data on maternal and fetal characteristics together with clinical data on pregnancy and delivery (Figueras et al. 2008). Gestational age was objectively determined for all registered births based on crown–rump length measurements (Robinson and Fleming 1975) from a routine ultrasound examination, usually during gestational weeks 11–13, or on biparietal diameter measurements made during weeks 13–22 (Mul et al. 1996).

We included singleton pregnancies with spontaneous onset of labor among mothers residing in Barcelona City. Although deliveries with cesarean section during labor were included in the analysis, multiple births (*n* = 150), elective (*n* = 553) and emergency (*n* = 282) cesarean sections, labor inductions (462), and mothers referred for obstetrical pathology (*n* = 159) were excluded.

**Figure 1:**
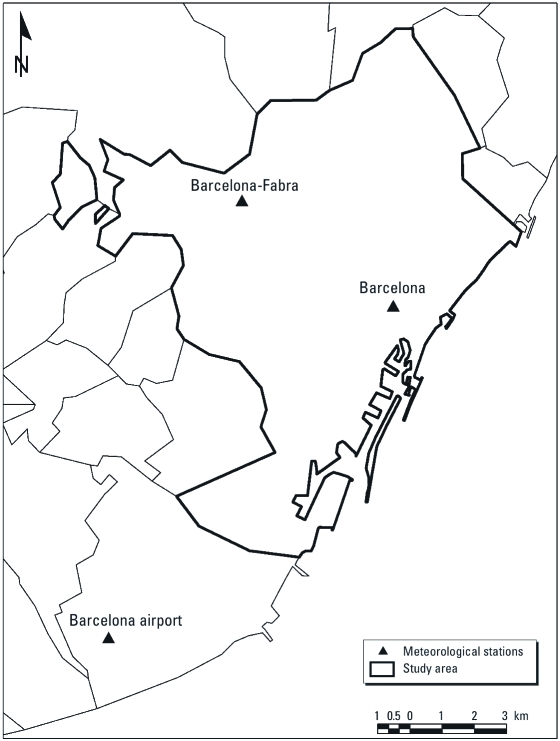
Meteorological monitoring sites across the Barcelona region, 1983–2006.

Barcelona is a port situated on the northeastern part of the Iberian Peninsula that has a Mediterranean climate, with hot and dry summers and mild winters (Catalunia Meteorological Service 2010). During the summer months (normally between May and October) Barcelona is affected by warm fronts originating from tropical and subtropical latitudes (Catalonia Meteorological Service 2010). Data on daily temperature and humidity for the period 1983–2006 were obtained from the Spanish meteorological agency (Agencia Estatal de Meteorología, Gobierno de España) and the Catalonia Meteorological Service (Servei Meteorològic de Catalunya) for three meteorological monitoring stations active across the region: Barcelona, Barcelona Airport, and Barcelona-Fabra stations, with elevations of 7, 6, and 412 m above sea level, respectively (Figure 1).

*Indicators of extreme heat conditions.* There is a lack of consensus on the definition of an extreme heat episode (Souch and Grimmond 2004). We therefore used three indicators of heat episodes based on 1-day exposure to an unusually high heat-humidity index: binary variables showing whether the heat index (HI) on each day has exceeded the 90th, 95th, or 99th percentile of heat indices (HI_90_, HI_95_, and HI_99_, respectively), derived from a historical series of 23 years (1983–2006). HI is an indicator of perceived temperature and is a function of both temperature and humidity calculated as follows (Kalkstein and Valimont 1986):

HI*_i_* = –2.653 + (0.994 × *Ta_i_*)   + (0.0153 × *Td_i_*^2^),

where *Ta_i_* is the average temperature for day *i* and *Td_i_* is the dew point for day *i*, calculated as


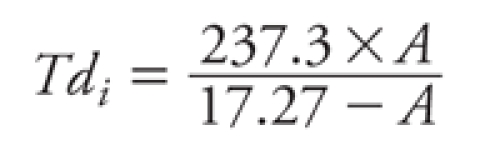



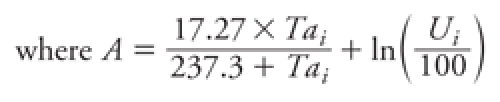


and *U_i_* is the relative humidity for day *i*. First, the HI was calculated for all days from 15 May to 15 October for the years 1983–2006 to abstract the 90th, 95th, and 99th percentiles (HI_90_, HI_95_, HI_99_). Then we created indicator variables indicating which days of the study period (2001–2005) exceeded those values.

In an attempt to address the duration of the heat episodes, we used the heat wave duration index [a binary variable indicating exceedance of the normal daily maximum temperatures by 5°C for > 5 consecutive days (Frich et al. 2002)] and identified the deliveries that occurred on the fifth day of the heat episode. It was not feasible to carry out analysis using this indicator because of the very small number of exposed subjects (*n* = 3).

*Exposure assignment.* The study area encompassed both sea-level plains and hillsides. The altitude of maternal places of residence ranged between zero and 508 m above sea level. We therefore calculated aerial distances to determine the closest monitoring station to maternal residences, accounting for elevation differences. A geographical information system (version 9.3; ArcGIS, ESRI Inc., Redlands, CA, USA) environment was used to extract the elevation difference (*e*) and distance (*d*) from all monitoring sites for each geocoded maternal address at the time of delivery. The aerial distance of each residential place to all monitoring sites was then calculated as √(*e*^2^ + *d*^2^). Exposures were determined for each subject based on measurements from the closest monitoring site according to the calculated aerial distances. HI indicators were then extracted for the delivery date (lag 0) and for lags of 1–6 days before the delivery date.

*Statistical analysis.* We used a two-stage analysis to estimate the impact of maternal exposure to extreme heat episodes on gestational age at delivery. The first stage was a dynamic model separating the monthly trend in gestational age at delivery for the study region as a whole from within-region personal variation. The second stage was a linear regression with individual gestational age at delivery as the outcome; a range of covariates, including indicators of extreme heat conditions, as predictors; and the predicted regional monthly trend by the first-stage model as an offset.

For the first stage, the following dynamic model was fitted to predict log-transformed regionwide monthly average of gestational age (µ^^^*_m_*) using the *sspir* library in the R statistical package (R Foundation for Statistical Computing, Vienna, Austria) (Dadvand et al. 2011; Fanshawe et al. 2007):

µ^^^*_m_* = α + β*m* + *B_m_*cos(ω*m*)   + *C_m_*sin(ω*m*) + *R_m_*, [1]

where α and β are static parameters, *B_m_* and *C_m_* are dynamic parameters, *m* is the corresponding month, ω is defined as 2π/12, and *R_m_* is the residual term for the month *m*. Specifically, *B_m_* and *C_m_* are modeled as random walks; hence, *B_m_* = *B_m_*_–1_ + *Z_m_* and *C_m_* = *C_m_*_–1_ + *W_m_*, where *Z_m_* and *W_m_* are time sequences of independent, zero-mean perturbations. The role of the random walk terms is to allow the amplitude and phase of the seasonal pattern to vary smoothly over time. The fitted model 1 [Equation 1] explained about 83% of variation in µ^^^*_m_* (*R*^2^ = 0.83), with little temporal autocorrelation in residuals. The second stage model was then fitted as

*Y_m_*(*x_i_*) = α + µ^^^*_m_*+ β*X* + *E_m_*(*x_i_*), [2]

where *Y_m_*(*x_i_*) is the gestational age (days) for subject *x_i_* delivered in month *m*, µ^^^*_m_* is the predicted regionwide monthly average of gestational age for month *m* in Equation 1, β and *X* are, respectively, the matrices of regression coefficients and predictors, and *E_m_*(*x_i_*) was the residual term for the subject *x_i_*. Model 2 (Equation 2) was repeated for lags 0–6 for the three HI indicators, resulting in 21 models. The analyses in model 2 were adjusted for maternal age (continuous) and categorical variables for ethnicity, academic level, work status, tobacco smoking, infection (rubella, group B streptococci, *Toxoplasma gondii*, sexually transmitted diseases, and/or bacteriuria), diabetes, history of preterm birth, history of obstetrical–gynecological pathologies (congenital uterine malformations, uterine myoma, congenital fetal malformation, and/or thrombophilic conditions), parity, use of assisted reproductive technique, and infant sex (Table 1).

**Table 1:** Covariates among the study subjects (7,585 births) used in model 2, Barcelona City, 2002–2005.

Covariate	*n* (%)*a*
Gestational age [weeks (mean ± SD)]	40 ± 1.9
Ethnicity	
European	4,401 (60.3)
South American	1,847 (25.3)
African	338 (4.6)
Asian	700 (9.6)
Oceanic	7 (0.1)
Maternal academic level	
No formal schooling	154 (2.3)
Primary school	1,805 (26.7)
Secondary school or professional college	3,057 (45.3)
University	1,737 (25.7)
Parity	
Nulliparous	4,501 (59.3)
1	2,288 (30.2)
2	567 (7.5)
≥ 3	228 (3.0)
Maternal history of preterm birth	
Yes	202 (2.7)
No	7,382 (97.3)
Use of assisted reproductive technique	
Yes	75 (1.0)
No	7,509 (99.0)
Maternal infection	
Yes	1,428 (18.8)
No	6,156 (81.2)
Maternal age [years (mean ± SD)]	30 ± 5.6
Maternal smoking during pregnancy	
Not smoking	6,218 (82.0)
1–9 cigarettes/day	767 (10.1)
10–19 cigarettes/day	451 (5.9)
> 19 cigarettes/day	148 (2.0)
Maternal occupational status	
Student or unemployed	1,647 (24.7)
Employee	4,453 (66.8)
Self-employed	565 (8.5)
Maternal diabetes	
No diabetes	7,028 (93.3)
Gestational diabetes	413 (5.5)
Pregestational diabetes	19 (0.3)
Glucose intolerance	73 (1.0)
Maternal obstetrical–gynecological pathology	
Yes	161 (2.1)
No	7,423 (97.9)
Infant sex	
Male	3,888 (51.3)
Female	3,695 (48.7)
**a**Data are *n* (%) except as noted.	

Self-identified data on maternal ethnicity with European, South American, African, Asian, and Oceania categories was used for this analysis. As a further step to explore the impact of maternal ethnicity on our investigated link, a subanalysis was limited to European mothers, and model 2 was repeated for this subset after removing ethnicity as a covariate. Although we were not able to carry out the analysis for other individual ethnic groups because of the very small number of exposed subjects, we compared European and non-European subjects by testing the interaction between HI_99_ episode and an European/non-European indicator.

*Ethical approval.* This study made use of anonymized administrative hospital data. The use of these data for this study was approved by the Clinical Research Ethical Committee of the Parc de Salut MAR, Barcelona, Spain (ethics approval no. 2010/3832/I).

## Results

During the course of the study, 9,191 births were registered in the cohort. Data from 7,585 births met the inclusion criteria for the study, including 44 stillbirths. Table 1 presents the distribution of the covariates among the study subjects. Gestational age at delivery ranged between 22.2 and 43.5 weeks, with an average of 40 weeks. For the period 1983–2006, the averages of 90th, 95th, and 99th percentiles of HI during warm months (15 May to 15 October) over the three meteorological stations were 29.1°C, 30.2°C, and 32.0°C, respectively (Table 2). HI_95_ and HI_99_ episodes mostly occurred during the June–August period, with peak in August for HI_99_ and in July for HI_90_ and H_95_.

**Table 2:** Maximum temperature and heat index (HI) recorded by the meteorological monitors across the city of Barcelona over the warm months (15 May to 15 October), 1983–2006.

Monitor	Maximum temperature (°C)													
	Range	90th percentile	95th percentile	99th percentile	HI (°C)						
					Range	HI_90_	HI_95_	HI_99_
Barcelona		13.8–37.3		27.9		28.6		30.4		10.7–36.4		29.6		30.5		32.0
Barcelona-Fabra		12.0–38.4		30.6		32.0		34.2		8.5–37.8		28.8		30.0		31.8
Barcelona Airport		13.7–35.7		29.8		30.6		33.0		10.2–35.1		29.0		30.2		32.3
Regionwide average		—		29.4		30.4		32.5		—		29.1		30.2		32.0

The average numbers of mothers experiencing HI_90_, HI_95_, and HI_99_ episodes on the day of delivery and the 6 days before delivery were 523, 221, and 32, respectively (Table 3). An HI_95_ episode on the day before the delivery day (lag 1) was associated with a 1.6-day reduction in the average length of pregnancy [95% confidence interval (CI), –3.5 to 0.2 days; *p* = 0.08], whereas an HI_95_ episode on the day of delivery was associated with a smaller reduction (–0.2 days; 95% CI, –0.4 to –0.1 day; *p* < 0.01) (Table 3). An HI_99_ episode on the day before delivery was associated with a 5-day reduction in gestational age at delivery (–5.3 days; 95% CI, –10.1 to –0.05 days; *p* = 0.03). The results for other lags were not conclusive.

**Table 3:** Adjusted*a* regression coefficients*b* for change in gestational age (days) due to maternal exposure to HI_90_, HI_95_, and HI_99_ episodes during lags of 0–6 days, Barcelona City, 2001–2005.

HI_90_					HI_95_					HI_99_				
Lag	No. of exposed subjects	Regression coefficient (95% CI)	*p*-Value	No. of exposed subjects	Regression coefficient (95% CI)	*p*-Value	No. of exposed subjects	Regression coefficient (95% CI)	*p*-Value
0		492		–0.7 (–1.9 to 0.6)		0.29		222		–0.2 (–0.4 to –0.1)		< 0.01		25		–1.1 (–6.3 to 4.1)		0.68
1		482		–0.9 (–2.1 to 0.4)		0.17		221		–1.6 (–3.5 to 0.2)		0.08		30		–5.3 (–10.1 to –0.5)		0.03
2		690		–1.0 (–2.1 to 0.0)		0.06		227		–1.2 (–3.1 to 0.6)		0.19		29		–1.7 (–3.5 to 6.9)		0.53
3		500		–0.8 (–2.1 to 0.4)		0.18		215		0.4 (–1.6 to 2.4)		0.71		27		–3.2 (–8.3 to 2.0)		0.23
4		491		0.2 (–1.0 to 1.5)		0.70		226		0.2 (–1.7 to 2.1)		0.84		37		–1.0 (–5.3 to 3.3)		0.65
5		508		0.1 (–1.2 to 1.3)		0.93		233		0.1 (–1.8 to 2.1)		0.91		36		–0.4 (–4.8 to 4.1)		0.87
6		502		–0.1 (–1.4 to 1.1)		0.82		205		0.8 (–1.2 to 2.7)		0.43		37		1.3 (–3.7 to 6.3)		0.61
**a**Adjusted for maternal age, ethnicity, academic level, work status, tobacco smoking, diabetes, infection, history of preterm birth, obstetrical–gynecological pathologies, parity, use of assisted reproductive technique, and infant sex. **b**Each regression coefficient represents results from a separate regression.																		

After limiting the analysis to Europeans, 292, 127, and 16 subjects were exposed to HI_90_, HI_95_, and HI_99_ episodes on the day before delivery. Average estimated reductions in the length of pregnancy associated with these episodes were, for HI_90_, –0.9 days (95% CI, –2.5 to 0.7 days; *p* = 0.28); for HI_95_, –1.7 days (95% CI, –4.0 to 0.6 days; *p* = 0.15); and for HI_99_, –8.9 days (95% CI, –15.5 to –2.2 days; *p* = 0.01). For non-Europeans, exposure to an HI_99_ episode on the day before delivery (*n* = 14) was associated with a decrease of –1.2 days (95% CI, –8.1 to 5.7 days; *p* = 0.73) in the average gestational age at delivery. The difference of these associations (HI_99_ episode on the day before delivery and gestational age at delivery) in Europeans and non-Europeans did not reach statistical significance level (*p* = 0.11 for the interaction term). For other HI_90_, HI_95_, and HI_99_ lags, we did not detect any clear association.

## Discussion

In this study we investigated the link between maternal short-term exposure to extreme ambient heat and gestational age at delivery, based on the results of an earlier small study showing an increase in preterm birth after short-term exposure to extreme ambient heat and humidity (Lajinian et al. 1997). We used prospectively collected data on maternal and fetal characteristics, including objectively measured gestational age, together with three indicators of extreme heat conditions to investigate this link.

Our study showed a small reduction (0.2 day) in average gestational age at delivery associated with an HI_95_ episode on the day of delivery that is not clinically important but is consistent with an immediate effect of exposure. In addition, while an HI_90_ episode on the day before delivery was associated with a 1-day reduction in average gestational age at delivery, a more extreme HI_95_ episode on the day before delivery was associated with a 2-day reduction, and the most extreme condition (HI_99_) was associated with a 5-day reduction, suggesting a dose–response relationship. We found little evidence of an effect related to longer lags, supporting an immediate effect of heat stress on pregnancy duration.

Some earlier studies have reported that heat stress can lead to uterine contractions in pregnant women (Khamis et al. 1983; Vähä-Eskeli and Erkkola 1991). Animal studies have shown an increased secretion of oxytocin and prostaglandin F_2α_ (PGF_2α_) due to heat stress (Dreiling et al. 1991; Rensis and Scaramuzzi 2003; Wolfenson et al. 1993). Both oxytocin and PGF_2α_ are known to be involved in the induction of labor in humans (Karim 1968; Kelly and Tan 2001; Kelly et al. 2009; Lee et al. 2008; Satoh et al. 1979). Heat stress also leads to the release of heat-shock proteins (HSPs), including HSP-70, in humans (Daugaard et al. 2007). Increased levels of HSP-70 have been linked to a range of adverse pregnancy outcomes, including preterm birth (Akimune et al. 2005; Hnat et al. 2005). This effect of HSP-70 is suggested to be due to induction of proinflammatory cytokines, leading to inflammation in maternal-fetal interface (Peltier 2003). Evidence of an immediate effect of heat stress from our study is consistent with these mechanisms (oxytocin, PGF_2α_, and HSP-70). Dehydration in extreme heat conditions could be another mechanism involved in shortening of gestation (Lajinian et al. 1997). However, we did not have information on maternal hydration status.

Heat stress has been suggested to have a role in the natural selection of body size and shape (Crognier 1981; Schreider 1964; Wells and Cole 2002). The ratio of body surface area to body mass, relative skin blood flow, and sweating capability are factors involved in mitigation of heat stress that vary among different ethnicities (Hanna and Brown 1983; Lambert et al. 2008; Millington and Wilkinson 1983; Wells and Cole 2002). Ethnically white people have been reported to have lower degrees of adaptation to heat stress compared with Africans (Baker 1958; Little and Haas 1989; Robinson et al. 1941; Wells 2002). There is also some evidence of ethnic variation in immune system genes, including *HSP-70* (Genc et al. 2004; Nguyen et al. 2004). Ethnic differences in the impact of maternal heat stress on pregnancy outcomes may provide clues for the underlying biological mechanisms. In our study, an HI_99_ episode on the day before delivery was associated with an 8-day reduction in average gestational age at delivery among Europeans, compared with a 5-day reduction for the population as a whole and a 1-day reduction for the non-Europeans.

We based our analysis on ambient measures of temperature and humidity. Indoor temperature and humidity levels would differ from ambient levels because of use of air conditioners, for instance. According to the 2001 census, 21% of households in Barcelona had air conditioners (Spanish National Institute of Statistics 2004). We were not able to address air conditioner use in our study because of unavailability of data, and this could have resulted in exposure misclassification.

## Conclusions

Shortening of pregnancy is not only related to perinatal mortality and morbidity but also linked with adverse outcomes in later life and would be therefore associated with a considerable personal and social burden. Future climate is predicted to have more frequent and intense extreme heat conditions. Experimental and animal studies support the biological plausibility of a causal link between maternal heat stress and shortening of pregnancy. However, the available epidemiological evidence for this link is surprisingly scarce. Based on objective measures of gestational age, our findings suggest a reduction in average gestational age at delivery after maternal exposure to extreme HI episodes. Replication in further studies is required to validate this finding and to be able to draw conclusions about this link and ultimately increase the scope for primary prevention. This is important considering the ubiquitous nature of this exposure and therefore the potential for considerable population-attributable risk due to this exposure. We could not investigate whether the association varied according to gestational age because of the small number of affected births, but future studies may want to explore the question to help properly target the interventions. We were also not able to investigate this link for ethnic groups other than Europeans. Finally, our indicators of exposure to extreme heat conditions did not take into account duration of the episodes. For future studies, we recommend addressing maternal time–activity patterns, hydration, and residential air conditioner use, as well as duration of the heat episodes, and stratifying the analysis according to the ethnicity in multiethnic populations.
